# The rise of neutron cryo-crystallography

**DOI:** 10.1107/S205979831800640X

**Published:** 2018-07-24

**Authors:** Hanna Kwon, Patricia S. Langan, Leighton Coates, Emma L. Raven, Peter C. E. Moody

**Affiliations:** a Henry Wellcome Laboratories for Structural Biology, Leicester Institute of Structural and Chemical Biology, Department of Molecular and Cell Biology, University of Leicester, Lancaster Road, Leicester LE1 7RH, England; b Neutron Scattering Division, Oak Ridge National Laboratory, Oak Ridge, TN 37831, USA; c Leicester Institute of Structural and Chemical Biology, Department of Chemistry, University of Leicester, University Road, Leicester LE1 7RH, England

**Keywords:** neutron crystallography, cryogenic data collection, enzyme mechanisms

## Abstract

The use of boiled-off liquid nitrogen to maintain protein crystals at 100 K during X-ray data collection has become almost universal. Applying this to neutron protein crystallography offers the opportunity to significantly broaden the scope of biochemical problems that can be addressed, although care must be taken in assuming that direct extrapolation to room temperature is always valid. Here, the history to date of neutron protein cryo-crystallography and the particular problems and solutions associated with the mounting and cryocooling of the larger crystals needed for neutron crystallography are reviewed. Finally, the outlook for further cryogenic neutron studies using existing and future neutron instrumentation is discussed.

## Abstract 2

The application of the cryogenic data-collection environments used in protein X-ray crystallography to neutron protein crystallography is discussed.

## 1. Introduction   

Macromolecular X-ray crystallography provides a wealth of three-dimensional structural information that greatly contributes to our understanding of protein function and provides insight into enzyme reaction mechanisms. The discovery by Dorothy Crowfoot (Bernal & Crowfoot, 1934 ▸) that protein crystals, when kept hydrated by being sealed in thin-walled glass capillaries along with their mother liquor, retain their crystalline order and thus could be used for X-ray diffraction experiments enabled protein crystallography. This method of mounting protein crystals remained the norm until the mid-1990s. Mounting crystals in this way meant that X-ray data collection could be carried out conveniently at room temperature. However, this method meant that any chemical reactions induced by X-rays would be propagated, resulting in damage. Cooling capillary-mounted crystals to below the freezing point of the mother liquor would cause ice-crystal formation, damaging the crystal and giving overwhelming ice diffraction. More modest cooling in the capillary (for example to 258 K with 2.8 *M* ammonium sulfate) to slow radiation damage (Skarżński *et al.*, 1987 ▸) and to catch labile catalytic intermediates (Moody, 1984 ▸) was not often employed and frequently produced problems with condensation. The susceptibility of crystals to radiation damage often meant that several crystals were needed to collect a complete data set, and each would need to be carefully aligned to avoid un­necessary duplication and waste. For small molecules that did not suffer from loss or change in phase of solvent, a boiled-off liquid-nitrogen apparatus was developed, allowing temperatures near 80 K to be used (Post *et al.*, 1951 ▸). The stability of protein crystals for long periods once sealed in capillaries and the lack of damage from photochemistry from neutron irradiation meant that this was the preferred method to mount protein crystals for neutron crystallography.

Pioneering studies on lactate dehydrogenase by Haas and Rossmann showed that the rate of radiation damage for cryocooled crystals is a factor of ten lower than for crystals at room temperature (Haas & Rossmann, 1970 ▸). The wide adoption of intense synchrotron radiation for X-ray protein crystallo­graphy meant that radiation-induced damage became a severe problem. Teng (1990 ▸) showed that it was possible to mount crystals in a loop with a minimal amount of mother liquor, and if the composition of that mother liquor was suitably adjusted then rapid cooling to temperatures around that of liquid nitrogen would prevent the formation of ice crystals, instead vitrifying the solvent to an amorphous glass. By maintaining the crystal in a stream of nitrogen gas at about 100 K, the X-ray-induced reactions damaging the crystal were slowed down sufficiently to often eliminate the need for multiple crystals for a single data set and furthermore resulted in better quality data. The elimination of scattering from the glass capillaries also reduced background noise. Higher X-ray doses could also be used, allowing data with improved resolution to be collected, and the slowing of the thermal motion (or disorder) of the protein molecules contributes to this. A derivation of this method became standard in the mid-1990s; the development of fast electronic detectors at synchrotron sources then required the development of robotic crystal-mounting/unmounting robots (Ohana *et al.*, 2004 ▸), and this meant that universal standards needed to be developed and applied (Cipriani *et al.*, 2006 ▸). These standards meant that crystals could be screened on the home source and then transported to any available synchrotron for data collection. These developments were largely ignored for neutron crystallography, as they seemed to be of little relevance. Why bother with the additional complications of cryogenic work when capillaries work well? After all, one advantage of using neutrons is that they do not cause any observable radiation damage to crystals. The much larger size of the crystals being used for neutron work also meant that the loops and supports developed for X-ray work were not always appropriate.

The idea of using cryogenic techniques to slow the chemistry of radiation damage can also be applied to other chemical reactions. The solvent content of a protein crystal is typically 20–80% (Matthews, 1968 ▸, 1976 ▸), meaning that there is often good access for substrates to the active site of the enzyme; furthermore, the inherent flexibility of this environment means that enzyme activity can be observed in the crystal. Thus, by careful manipulation of conditions it is sometimes possible to form interesting intermediate states in the crystal and then trap these under cryogenic conditions for crystallo­graphic structure determination. Microspectrophotometry can often be used to monitor the progress of reactions in the crystal. However, it is important to be aware that rapid cooling to 100 K does not simply fix the motion of the room-temperature structure, as water undergoes glass transitions at 136 and 116 K (Amann-Winkel *et al.*, 2013 ▸). One of the early attempts to use X-ray cryo-crystallography to trap intermediates was to characterize two of the functionally relevant intermediate states on the reaction pathway of the ubiquitous haem-containing monooxygenase called cytochrome P450. Unstable reaction intermediates of cytochrome P450 were prepared using this trapping technique. The structures of the oxyferryl intermediates obtained by X-ray cryo-crystallo­graphy showed conformational changes in several important residues and revealed a network of bound water molecules (Schlichting *et al.*, 2000 ▸). These intermediate structures provided valuable information for understanding the P450 reaction mechanisms.

However, X-rays are strongly ionizing and photoreduction will compromise the information recovered, even under cryo-conditions. Examples of photoreduction altering the chemical nature of cryo-trapped intermediates are given in Fülöp *et al.* (1994 ▸), Bonagura *et al.* (2003 ▸) and Gumiero *et al.* (2011 ▸), and are discussed in Kwon *et al.* (2017 ▸). X-rays in crystals are scattered by the atomic electrons, and the combination of low (or absent) electron density for H atoms and the short distance (∼1 Å) to neighbouring ‘heavy’ atoms makes the positions or presence of H atoms very difficult to determine with confidence, even in the cases (<1% of the PDB) where subatomic resolution data are available. Without information about protonation states, critical information towards the understanding of the mechanisms of enzyme catalysis is lost. Neutron crystallography provides an alternative approach to determining the H-atom positions. As the coherent neutron scattering length of deuterium (an isotope of hydrogen) is similar to those of heavier atoms such as C, O and N, the positions of hydrogen and its presence can be located at much lower resolution (2.0–2.5 Å) than the subatomic resolution required by X-rays; furthermore, neutrons do not cause any observable radiation damage to crystals.

## 2. Neutron cryo-crystallography   

Although the collection of neutron data at cryo-temperatures is not normally required to extend the lifetime of the crystal in the beam, it does have particular uses and advantages. The lack of radiation damage and the much more detailed description of the water structure means that neutron crystallography provides an opportunity to properly compare room-temperature and cryo structures. As for X-rays, cryo-cooling of the crystals can reduce atomic movement, thus improving the resolution. The application for neutrons of the techniques used in X-ray work for trapping catalytic intermediates allows the proton (or deuteron) positions to be seen, greatly enhancing the mechanistic information available. Cryogenic data collection allows the study of any proteins and protein complexes (including those with ligands and nucleic acids) that are not stable enough at room temperature for the prolonged data-collection period required for neutron crystallography. To date, very few neutron structures at cryogenic temperatures have been deposited in the Protein Data Bank (PDB). The first pioneering low-resolution neutron cryogenic neutron work was conducted on myoglobin, studying the solvent structure as a function of temperature (Daniels *et al.*, 1996 ▸). A further low-resolution cryogenic structure of myoglobin was published the following year at a resolution of 5 Å (Daniels *et al.*, 1997 ▸). These early studies focused on the hydration layers surrounding the protein in the crystal.

The other cryogenic neutron structures deposited in the PDB are of saccharide-free concanavalin (Blakeley *et al.*, 2004 ▸), cytochrome *c* peroxidase (Casadei *et al.*, 2014 ▸), Toho1 β-lactamase in complex with an inhibitor (Coates *et al.*, 2014 ▸), T4 lysozyme (Li *et al.*, 2017 ▸) and ascorbate peroxidase (Kwon *et al.*, 2016 ▸). These structures have highlighted the potential of cryogenic neutron diffraction for certain types of studies.

The first medium-resolution neutron structure collected at cryo-temperature (using a Displex cryorefrigerator to 15 K) was that of apo concanavalin A (Con A) at 2.50 Å resolution (Blakeley *et al.*, 2004 ▸). Con A is a saccharide-binding protein that belongs to the well studied legume lectin family. Con A contains two metal-binding sites which must be occupied for the protein to bind saccharide. Detailed comparison with neutron structures at room temperature (Habash *et al.*, 2000 ▸) and 15 K allowed analysis of the structural changes upon cryocooling. The 15 K structure showed twice as many water molecules bound to the protein compared with the room-temperature structure, and the orientations of many waters found in both the 15 K and the room-temperature structures were different. In addition, the *B* factors for the waters were reduced by fourfold in the 15 K structure. The 100 K Toho1 β-lactamase structure gave improved nuclear density for a transiently bound ligand which binds at the active site, allowing the protonation state of a nearby glutamate residue to be more clearly determined. Cryogenic neutron diffraction studies of cytochrome *c* peroxidase resolved a decades-old debate by revealing that the ferryl haem in compound I is unproton­ated, and the neutron structure of the ferryl intermediate in compound II of ascorbate peroxidase allows the protonation states of the ferryl haem to be directly observed. The neutron structure of T4 lysozyme revealed in detail the hydrogen-bonding interactions within the protein, such as the atomic nature of the salt bridge between His31 and Asp70.

It was known from X-ray work that some changes in solvent position and protein conformation are seen when cryocooled structures are compared with room-temperature structures. Upon cooling, the unit cell will shrink, possibly altering the crystallographic contacts. The vitrification process will freeze out side-chain motion, and this may not be in the conformation that is predominantly adopted at room temperature. The water mobility may also affect and be affected by the restriction of protein conformational freedom. An example is that of Keedy *et al.* (2014 ▸), who produced a detailed study comparing independently acquired pairs of X-ray data sets of a dihydrofolate reductase (DHFR) complex collected at both room temperature and 100 K (Keedy *et al.*, 2014 ▸). This allowed some confidence that the changes were consistent rather than just variations within samples. They noted that the dual conformations of some side chains in the room-temperature structure are reduced to a single conformer in the 100 K structures. Temperature changes can also alter the conformations of amino-acid residues that interact with the bound substrate molecules. A particular example is the side chain of Arg52, which is positioned next to the folate substrate whilst also being at a crystal lattice interface. Upon cryocooling (to 100 K) the local energy landscape changes. This causes the side chain of Asp87 on an adjacent molecule to switch rotamers and thus point away from Arg52, allowing Arg52 to occupy this space with a rotamer that is not seen at all in the room-temperature structure. This results in a new salt bridge between Arg52 and Asp70 of an adjacent protein molecule. Thus, in the room-temperature structure Arg52 is placed near the folate substrate, but is not seen in the 100 K structures where the electrostatic interaction between the NH_2_ group of Arg52 and the folate substrate is substituted by a water molecule.

**Figure 1:**
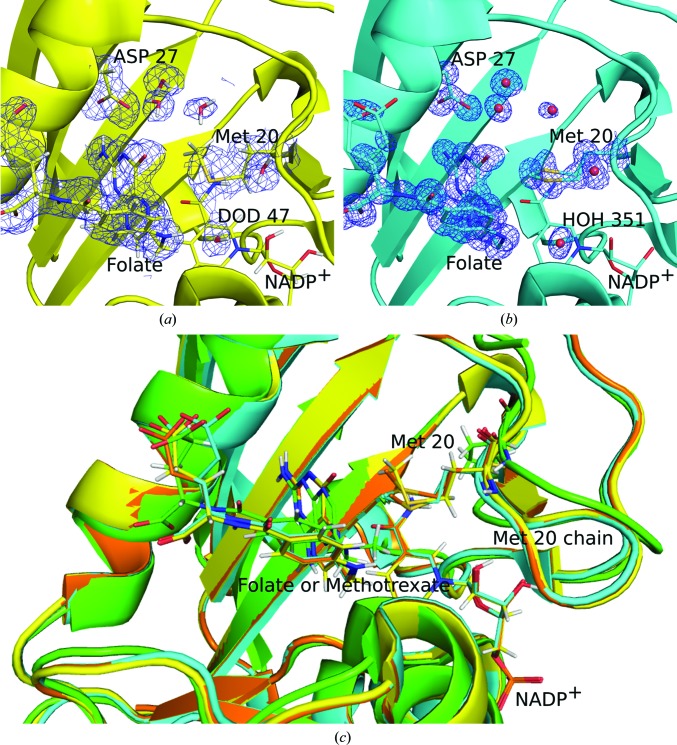
The neutron structure of DHFR in complex with folate and NADP^+^. (*a*) The neutron structure (Wan, Kovalevsky *et al.*, 2014 ▸; Wan, Bennett *et al.*, 2014 ▸). N atoms are shown in blue, C atoms in yellow, S atoms in gold and O atoms in red. Nuclear density 2*F*_o_ − *F*_c_ maps are represented at a σ level of 0.8. (*b*) The X-ray structure collected at 100 K (Wan, Kovalevsky *et al.*, 2014 ▸). C atoms are shown in cyan and water molecules are represented by red spheres. Electron density 2*F*_o_ − *F*_c_ maps are represented at a σ level of 1.2. (*c*) Structure alignment of the neutron structure (Wan, Bennett *et al.*, 2014 ▸), the 100 K X-ray structure (Wan, Kovalevsky *et al.*, 2014 ▸), the 277 K X-ray structure (Wan, Bennett *et al.*, 2014 ▸; C atoms represented in orange) and the neutron structure of DHFR in complex with methotrexate (Bennett *et al.*, 2006 ▸; C atoms represented in green). For clarity, density is shown only for certain active-site residues and ligands.

Neutron structures of DHFR in complex with methotrexate (Bennett *et al.*, 2006 ▸) and in complex with folate and NADP^+^ (Wan, Bennett *et al.*, 2014 ▸) were both determined at room temperature. These have given further insights into the catalytic mechanism of DHFR and the mobility of the protein. Because both samples were expressed and grown under hydrogenated conditions and then underwent hydrogen/deuterium (H/D) exchange after crystallization, the mobility and accessibility of various regions of the protein could be further quantified (Bennett *et al.*, 2006 ▸; Wan, Bennett *et al.*, 2014 ▸). This was particularly useful for the Met20 loop, which adopted a closed conformation in all DHFR–folate structures (100, 277 and 291 K X-ray structures and the 291 K neutron structure), with both a closed and open Met20 loop being present in the DHFR–methotrexate complex (293 K neutron structure; Fig. 1 ▸ *c*). Despite the lack of solvent accessibility in the closed conformation, the Met20 loops in all of these structures showed complete or almost complete H/D exchange, indicating that this is a highly dynamic region and supporting the mobility discussed in the paper by Keedy and coworkers (Keedy *et al.*, 2014 ▸; Bennett *et al.*, 2006 ▸; Wan, Bennett *et al.*, 2014 ▸). Additionally, despite having a closed loop conformation which should preclude a water molecule being within hydrogen-bonding distance of the folate N10 atom, there is density for a partially occupied water molecule within hydrogen-bonding distance of this atom. This partially occupied water is seen in both the room-temperature neutron structure (DOD47) and 100 K X-ray structure (HOH351) of the DHFR–folate complex (Fig. 1 ▸ *a*). As this water molecule is within hydrogen-bonding distance of the folate N10 atom, it could be responsible for protonation of N5 of the bound folate molecule.

Further examination of the active site of the neutron structure of the DHFR–folate complex showed a proton on the N3 atom of folate and a negatively charged Arg27. Along with the partially occupied water molecule near the folate N10 atom, these results indicate direct protonation of the substrate by the solvent and dismiss tautomerization as a necessity for catalysis (Wan, Bennett *et al.*, 2014 ▸).

## 3. Cryogenic data collection at neutron facilities   

Although cryogenic data-collection capabilities have been available at previous generations of neutron protein crystallography (NPX) instruments, for example, use of the cryogenic data-collection facilities at the recently decommissioned Protein Crystallography Station (PCS) at Los Alamos National Laboratory (Schoenborn & Langan, 2004 ▸; Langan *et al.*, 2008 ▸) was never reported during its 15 years of operation (Chen & Unkefer, 2017 ▸). One possible reason for this is the difficulty associated with successfully cryocooling the larger crystals needed for neutron diffraction and maintaining them at 100 K over the many days required to collect a complete neutron data set. More recently, the deployment of equipment and facilities for 100 K cryogenic neutron data collection has been a common theme in upgrades at NPX instruments around the world (Coates *et al.*, 2014 ▸, 2015 ▸). Although NPX instruments at spallation and reactor sources use significantly different instrument designs and detector technologies, they share several characteristics and it is worth reviewing them here. Owing to the lower flux and longer wavelengths used for neutron diffraction compared with X-ray diffraction, a large solid-angle detector coverage is highly desirable to reduce the number of crystal orientations needed for a complete data set. Therefore, a nitrogen cryostream is often positioned above a vertically mounted goniostat and rotation axis such that the sample mount makes an angle close to 180° with the cryostream. This has been found to help to reduce ice formation on the sample pin during the extended time required for neutron data collection. The incoming neutron beam is then horizontal to the sample position to form a 90° angle with the cryostream. One benefit of large angular detector coverage is that the detectors and the infrastructure used to support them often help to isolate the sample from the surrounding atmosphere, preventing drafts and humid air from reaching the sample position. The upgraded LADI-III instrument (Blakeley *et al.*, 2010 ▸) at the at the Institut Laue–Langevin, the iBIX instrument at the Japanese Particle Research Accelerator Complex (J-PARC; Kusaka *et al.*, 2013 ▸) and the BIODIFF instrument at the FRM II research reactor have deployed similar equipment for cryogenic neutron data collection (Coates *et al.*, 2014 ▸), which has been utilized in recent studies (Casadei *et al.*, 2014 ▸; Kwon *et al.*, 2016 ▸). A different approach using a liquid-helium Displex cryorefrigerator is also possible. This was used originally on LADI-I (Myles *et al.*, 2012 ▸; retained as a 15 K option on LADI-III) and has recently been deployed on the IMAGINE instrument (Meilleur *et al.*, 2013 ▸). It enables lower temperatures to be reached (4 K) but requires the use of an aluminium container that blocks direct viewing of the sample, complicating the alignment of the crystal into the neutron beam. The cooling of the crystal by conduction also prevents the use of standard X-ray mounts. However, it does enable different areas of science besides NPX to be served by the same instrument.

**Figure 2:**
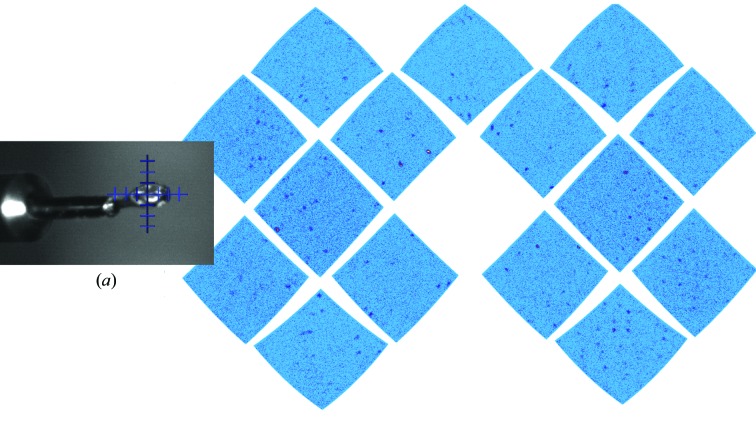
(*a*) A cryocooled perdeuterated crystal of Toho1 β-lactamase in complex with the antibiotic cefotaxime, freeze-trapping a short-lived intermediate over a 40 h data-collection time. The crystal volume is around 0.5 mm^3^ and it is mounted in a specially made 2 mm diameter litholoop from Molecular Dimensions. The large crystal fits snugly into the custom-sized loop, making it less likely that the crystal will be lost during the cryocooling process. (*b*) A time-of-flight wavelength-resolved slice from one of five 8 h diffraction images from a Toho1 β-lactamase data collection on the MaNDi instrument.

On the macromolecular neutron diffractometer (MaNDi) at the Spallation Neutron Source (SNS) at Oak Ridge National Laboratory (ORNL) the spherical detector-array frame has an internal diameter of around 70 cm, which completely encloses the sample position when the goniometer is in place, thus isolating the sample from the large air volume within the MaNDi instrument hutch (Coates *et al.*, 2015 ▸), while on reactor-based NPX instruments the most often used detector is a cylindrical arrangement of a specially treated neutron image plate around the sample mount, both with coincident vertical axes of rotation. The readout of these image-plate drum detectors (LADI-III, BIODIFF and IMAGINE) requires rotation of the image-plate drum, which creates air turbulence that can interfere with the cryostream dry-gas flow and cause ice buildup to occur on the sample. To mitigate this issue, an aluminium shroud is often placed around the sample to stop the flow from being disturbed during readout. As the readout of the detectors on MaNDi does not require movement, data can be collected without the aluminium shroud. An example diffraction image from a Toho1 β-lactamase crystal collected on the MaNDi instrument at 100 K is shown in Fig. 2 ▸. β-Lactamases are troublesome enzymes which have evolved specifically to breakdown β-lactam antibiotics (Drawz & Bonomo, 2010 ▸). They are the leading cause of resistance to this class of potent and widely used antibiotics and as such are a major health problem, increasing surgical complications and mortality rates (Livermore & Woodford, 2006 ▸). Several neutron structures of Toho1 β-lactamase have been published that have studied the role of the catalytic residue Glu166 in the acylation reaction (Tomanicek *et al.*, 2013 ▸), while later studies probed the role of the catalytic residue Lys73 in the opening of the β-lactam ring (Vandavasi *et al.*, 2016 ▸, 2017 ▸) and the changes that occur upon substrate binding (Langan *et al.*, 2018 ▸). However, even engineered mutants of β-lactamase enzymes in which the catalytic residues are mutated still show activity against several types of antibiotics and β-lactamase inhibitors (Nitanai *et al.*, 2010 ▸; King *et al.*, 2015 ▸). Many β-lactamase inhibitors form a covalent interaction with the catalytic nucleophile Ser70 that is reversible in a period of around 24 h (King *et al.*, 2015 ▸). Therefore, to study the inter­actions between Toho1 β-lactamase and these types of antibiotics and inhibitors, it is necessary to collect data at 100 K just after the ligand has been soaked into the β-lactamase crystal (Fig. 2 ▸). The data statistics for the data collection shown in Fig. 2 ▸ are given in Table 1 ▸.

The facility to collect neutron data in a manner analogous to X-ray data collections at 100 K means that standard cryopins and loop-mounting systems can be used (Coates *et al.*, 2014 ▸). This means that the same crystal can be transferred to an X-ray setup to facilitate joint refinement. It also means that the crystal can be examined using the spectroscopic techniques developed for single crystals (von Stetten *et al.*, 2015 ▸). In the future, it may be possible to employ the robotic sample changers developed for synchrotron beamlines and automated screening at neutron crystallography stations. However, the crystals used for neutron diffraction are often larger and care must be taken to ensure successful cryocooling. Incoherent scattering from the H atoms within a sample is the main source of experimental background in an NPX experiment (Blakeley, 2009 ▸), therefore it is advisable to utilize a perdeuterated cryoprotectant if possible. This will reduce the background significantly, but other sources of background such as air scattering and the detection of neutrons not scattered by the sample will still remain. Fully perdeuterated glycerol, 2-methyl-2,4-pentanediol (MPD) and trehalose are all now commercially available; however, they are significantly more expensive than their hydrogenated versions and thus should be used in small volumes. Careful matching of the loop size to just a little larger than the crystal volume reduces the volume of vitreous mother liquor in the neutron beam, further reducing incoherent scattering from the sample. Larger crystals have a tendency to be dislodged from smaller loops, which they tend to sit on during the cryocooling process, and can often pick up ice during data collection (Coates *et al.*, 2014 ▸). Therefore, it is advisable to use a larger loop size for mounting and cooling large crystals (Fig. 2 ▸ *a*). Unfortunately, loops sized above 1 mm are generally not currently commercially available; however, litholoops with custom loop sizes of 2 and 3 mm diameter have been fabricated by Molecular Dimensions and are available to users of the MaNDi instrument. Other techniques such as the SPINE-compatible laser-cut microshaped vitreous carbon sample mounts have been developed (Romoli *et al.*, 2014 ▸) and are also available in custom sizes. The lessons learned from cryogenic protein X-ray crystallography need to be applied, with the added complication that as the surface area to volume ratio is likely to be lower for larger crystals, the effective cooling rate will be reduced. Because of this, following good practice for successful cryocooling is critical (Garman & Schneider, 1997 ▸). Prior to cryocooling, the crystal must be gently acclimatized to the composition of the cryoprotectant mother liquor otherwise the environmental changes upon transferring a crystal are likely to degrade its quality. For hydrogenated crystals this also means that a thorough H/D exchange should be completed, with an appropriate correction for pH/pD differences, before cryocooling is attempted. For the Toho1 β-lactamase crystals shown in Fig. 2 ▸, immersion into a dewar overfilled with liquid nitrogen seemed to be the most repeatable method of successful cryocooling. The overfilling of the dewar to the brim reduces or eliminates the vapour layer that collects on top of liquid nitrogen (Garman & Schneider, 1997 ▸). Direct cryocooling on the instrument cryostream has also been successful (Coates *et al.*, 2014 ▸) but several crystals were lost during the process. One drawback of this direct cooling technique is that mounting in a loop requires a microscope and several solutions close to the sample position of the diffractometer, resulting in a crowded environment that can be awkward for rapid and efficient transfer. Other techniques, such as immersion in liquid propane (Teng & Moffat, 1998 ▸) may be useful for cooling larger crystals, although further work is needed to determine whether the extra hazards presented by the handling of liquid propane are offset by the improved crystal quality after cryocooling.

Neutron diffraction often utilizes Laue (or quasi-Laue) techniques coupled with a high beam divergence to increase flux on the sample. This, combined with an increase in the mosaicity of the sample caused by cryocooling (Garman & Schneider, 1997 ▸), increases the size and range of the Bragg reflections (Fig. 2 ▸ *b*). As a result, it is difficult to successfully collect sufficient data from samples with larger unit cells owing to reflection overlap. However, this can be partially offset on wavelength-resolved time-of-flight neutron diffraction instruments, whereby refection density is significantly reduced by the ability to select monochromatic slices (Schoenborn & Langan, 2004 ▸; Coates & Robertson, 2017 ▸).

## 4. New science from neutrons at 100 K: haem peroxidases   

Haem peroxidases catalyse the reduction of peroxide to water, using high-oxidation-state intermediates (compound I and compound II) to activate peroxide and oxygen. It has been difficult to study this activation using X-ray crystallography as the photoelectrons generated during X-ray exposure reduce many of the chemically interesting high-valence species. In order to understand the mechanisms of the peroxidase, it is necessary to see the protonation states of these intermediates (Fe=O or Fe—OH). Early work on haem peroxidases using X-ray crystallography and various spectroscopic methods reported varying results on the identity of the haem ligand extrapolated from bond order based on the Fe—O distances, rather than direct visualization, owing to the limitations of X-ray crystallography (Fülöp *et al.*, 1994 ▸; Bonagura *et al.*, 2003 ▸; Gumiero *et al.*, 2011 ▸). These distances were difficult to establish with confidence, as well as the strongly electropositive iron being susceptible to reduction by photoelectrons (an effect often ignored in these and other redox enzymes). Furthermore, the close proximity of a relatively electron-dense Fe atom and a lighter O atom means that series-termination errors in the Fourier transform are likely to distort the distance between these atoms (Fülöp *et al.*, 2000 ▸). When the data are incomplete, of low resolution or of poor quality, and if the models are incomplete or have high *B* values, the uncertainties in atomic positions are also increased (Murshudov & Dodson, 1997 ▸). This difficulty in measuring the distances with the accuracy required (∼0.2 Å) meant that it was difficult to identify the ligand species conclusively.

**Figure 3:**
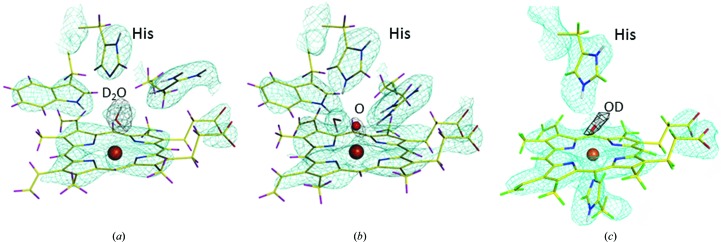
The structures of the intermediates of haem peroxidases. Nuclear density is shown in green and difference density for the ligand is shown in black. (*a*) The ferric (resting) enzyme cytochrome *c* peroxidase, (*b*) compound I of cytochrome *c* peroxidase, (*c*) compound II of ascorbate peroxidase.

**Figure 4:**
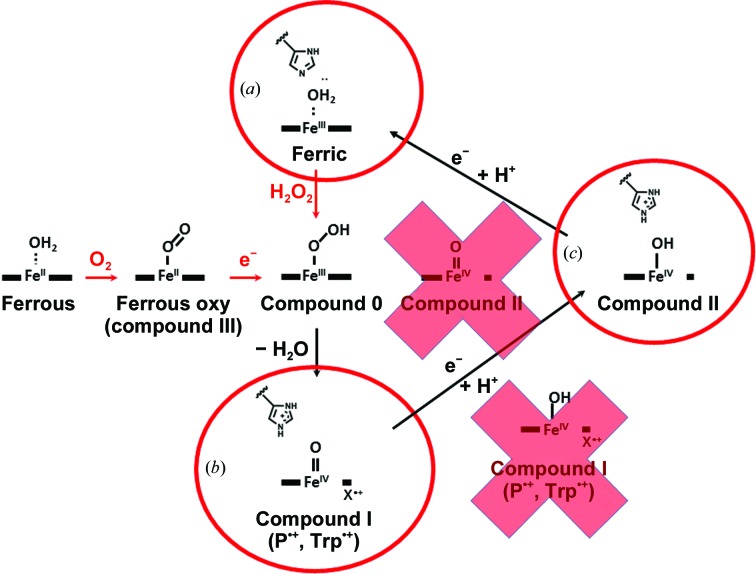
The intermediates in the reaction of haem peroxidases. The structures determined by NPX are circled. (*a*) The ferric (resting) enzyme from cytochrome *c* peroxidase, (*b*) compound I of cytochrome *c* peroxidase, (*c*) compound II of ascorbate peroxidase. The radicals on the porphyrin in the case of ascorbate peroxidase and on a tryptophan residue in the case of cytochrome *c* peroxidase are shown as 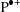 and 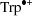, respectively. The red crosses show alternative structures eliminated by NPX.

Neutron cryo-crystallography provided answers to both problems. NPX meant there would be no photoreduction/radiation damage. In addition, atoms can be observed directly. With the newly introduced cryo setups at many neutron reactors, as mentioned above, the intermediate states can be trapped in the crystals and maintained for the duration of the long data-collection time. Recently, neutron structures of compound I of cytochrome *c* peroxidase (Casadei *et al.*, 2014 ▸) and compound II of soybean ascorbate peroxidase (Kwon *et al.*, 2016 ▸) have been solved (Fig. 3 ▸). In these experiments, the intermediates were formed by reaction in the crystals and the reaction was followed by spectroscopic means. The identity and longevity of the intermediates in the crystals at 70–100 K was verified by EPR. By trapping the intermediates and collecting neutron data at 100 K it was possible to show directly that cytochrome *c* peroxidase compound I was the Fe=O form and that the catalytic histidine was unexpectedly protonated (Casadei *et al.*, 2014 ▸), and that compound II of ascorbate peroxidase is Fe—OH (Kwon *et al.*, 2016 ▸). The recent ability to combine cryo-trapping of enzyme intermediates with neutron crystallo­graphy opens up the possibility of gaining a whole new level of insight into enzyme mechanism (Fig. 4 ▸).

## 5. Concluding remarks   

The first cryogenic NPX experiments were conducted in the 1990s (Daniels *et al.*, 1996 ▸) at low resolution, followed by a 15 K structure in 2004 (Blakeley *et al.*, 2004 ▸). In the last four years the collection of cryogenic neutron data has become more feasible in part owing to improved sources and instrumentation, which enable faster data-collection times. Perdeuteration of the protein increases the scattering power of the crystal and reduces the background emanating from the sample, enabling the use of smaller crystals for data collection. Small crystals are more easily cryocooled successfully and cryogenic data collection will become more routine as more advanced and powerful NPX instruments are constructed such as the Ewald instrument at the second target station at the SNS (Coates & Robertson, 2017 ▸) and the NMX diffractometer at the European Spallation Source. The lack of radiation damage by neutrons makes them ideal for collecting data at room temperature, but for a subset of experiments the ability to freeze-trap a short-lived intermediate enables NPX to address interesting scientific questions.

## Figures and Tables

**Table 1:** Data-collection statistics for a 100 K data set for Toho1 β-lactamase in complex with the antibiotic cefotaxime

Wavelength(s) (Å)	2–4
Temperature (K)	100
Detector(s)	40 SNS Anger cameras
Crystal-to-detector distance (mm)	450
No. of images collected	5
Total rotation range (°)	100
Exposure time per image (h)	8
Space group	*P*3_2_21
*a*, *b*, *c* (Å)	73.74, 73.74, 99.66
α, β, γ (°)	90, 90, 120
Resolution range (Å)	14.82–2.30 (2.38–2.30)
Total No. of reflections	28772
No. of unique reflections	11744
Completeness (%)	81.29 (80.60)
Multiplicity	2.45 (2.12)
〈*I*/σ(*I*)〉	5.5 (3.80)
*R*_merge_ (%)	20.1 (21.40)
*R*_p.i.m._ (%)	12.60 (15.40)

## References

[bb1] Amann-Winkel, K., Gainaru, C., Handle, P. H., Seidl, M., Nelson, H., Böhmer, R. & Loerting, T. (2013). *Proc. Natl Acad. Sci. USA*, **110**, 17720–17725. 10.1073/pnas.1311718110 PMC3816484 24101518

[bb2] Bennett, B., Langan, P., Coates, L., Mustyakimov, M., Schoenborn, B., Howell, E. E. & Dealwis, C. (2006). *Proc. Natl Acad. Sci. USA*, **103**, 18493–18498. 10.1073/pnas.0604977103 PMC1664550 17130456

[bb3] Bernal, J. D. & Crowfoot, D. (1934). *Nature (London)*, **133**, 794–795.

[bb4] Blakeley, M. P. (2009). *Crystallogr. Rev.* **15**, 157–218.

[bb6] Blakeley, M. P., Kalb, A. J., Helliwell, J. R. & Myles, D. A. A. (2004). *Proc. Natl Acad. Sci. USA*, **101**, 16405–16410. 10.1073/pnas.0405109101 PMC534515 15525703

[bb7] Blakeley, M. P., Teixeira, S. C. M., Petit-Haertlein, I., Hazemann, I., Mitschler, A., Haertlein, M., Howard, E. & Podjarny, A. D. (2010). *Acta Cryst.* D**66**, 1198–1205. 10.1107/S0907444910019797 21041937

[bb8] Bonagura, C. A., Bhaskar, B., Shimizu, H., Li, H., Sundaramoorthy, M., McRee, D. E., Goodin, D. B. & Poulos, T. L. (2003). *Biochemistry*, **42**, 5600–5608. 10.1021/bi034058c 12741816

[bb9] Casadei, C. M., Gumiero, A., Metcalfe, C. L., Murphy, E. J., Basran, J., Concilio, M. G., Teixeira, S. C. M., Schrader, T. E., Fielding, A. J., Ostermann, A., Blakeley, M. P., Raven, E. L. & Moody, P. C. E. (2014). *Science*, **345**, 193–197. 10.1126/science.1254398 25013070

[bb10] Chen, J. C.-H. & Unkefer, C. J. (2017). *IUCrJ*, **4**, 72–86. 10.1107/S205225251601664X PMC5331467 28250943

[bb11] Cipriani, F. *et al.* (2006). *Acta Cryst.* D**62**, 1251–1259. 10.1107/S0907444906030587 17001102

[bb12] Coates, L., Cuneo, M. J., Frost, M. J., He, J., Weiss, K. L., Tomanicek, S. J., McFeeters, H., Vandavasi, V. G., Langan, P. & Iverson, E. B. (2015). *J. Appl. Cryst.* **48**, 1302–1306.

[bb14] Coates, L. & Robertson, L. (2017). *J. Appl. Cryst.* **50**, 1174–1178. 10.1107/S1600576717010032 PMC5541355 28808436

[bb15] Coates, L., Tomanicek, S., Schrader, T. E., Weiss, K. L., Ng, J. D., Jüttner, P. & Ostermann, A. (2014). *J. Appl. Cryst.* **47**, 1431–1434.

[bb16] Daniels, B. V., Schoenborn, B. P. & Korszun, Z. R. (1996). *Basic Life Sci.* **64**, 325–331. 10.1007/978-1-4615-5847-7_28 9031517

[bb17] Daniels, B. V., Schoenborn, B. P. & Korszun, Z. R. (1997). *Acta Cryst.* D**53**, 544–550. 10.1107/S0907444997004356 15299885

[bb18] Drawz, S. M. & Bonomo, R. A. (2010). *Clin. Microbiol. Rev.* **23**, 160–201. 10.1128/CMR.00037-09 PMC2806661 20065329

[bb19] Fülöp, V., Phizackerley, R. P., Soltis, S. M., Clifton, I. J., Wakatsuki, S., Erman, J., Hajdu, J. & Edwards, S. L. (1994). *Structure*, **2**, 201–208. 10.1016/s0969-2126(00)00021-6 8069633

[bb20] Fülöp, V., Watmough, N. J. & Ferguson, S. J. (2000). *Adv. Inorg. Chem.* **51**, 163–204.

[bb21] Garman, E. F. & Schneider, T. R. (1997). *J. Appl. Cryst.* **30**, 211–237.

[bb22] Gumiero, A., Metcalfe, C. L., Pearson, A. R., Raven, E. L. & Moody, P. C. E. (2011). *J. Biol. Chem.* **286**, 1260–1268. 10.1074/jbc.M110.183483 PMC3020733 21062738

[bb23] Haas, D. J. & Rossmann, M. G. (1970). *Acta Cryst.* B**26**, 998–1004. 10.1107/s0567740870003485 5536135

[bb24] Habash, J., Raftery, J., Nuttall, R., Price, H. J., Wilkinson, C., Kalb (Gilboa), A. J. & Helliwell, J. R. (2000). *Acta Cryst.* D**56**, 541–550. 10.1107/s0907444900002353 10771422

[bb25] Keedy, D. A., van den Bedem, H., Sivak, D. A., Petsko, G. A., Ringe, D., Wilson, M. A. & Fraser, J. S. (2014). *Structure*, **22**, 899–910. 10.1016/j.str.2014.04.016 PMC4082491 24882744

[bb26] King, D. T., King, A. M., Lal, S. M., Wright, G. D. & Strynadka, N. C. J. (2015). *ACS Infect. Dis.* **1**, 175–184. 10.1021/acsinfecdis.5b00007 27622530

[bb27] Kusaka, K., Hosoya, T., Yamada, T., Tomoyori, K., Ohhara, T., Katagiri, M., Kurihara, K., Tanaka, I. & Niimura, N. (2013). *J. Synchrotron Rad.* **20**, 994–998. 10.1107/S0909049513021845 PMC3795571 24121355

[bb29] Kwon, H., Basran, J., Casadei, C. M., Fielding, A. J., Schrader, T. E., Ostermann, A., Devos, J. M., Aller, P., Blakeley, M. P., Moody, P. C. E. & Raven, E. L. (2016). *Nature Commun.* **7**, 13445. 10.1038/ncomms13445 PMC5141285 27897163

[bb30] Kwon, H., Smith, O., Raven, E. L. & Moody, P. C. E. (2017). *Acta Cryst.* D**73**, 141–147. 10.1107/S2059798316016314 PMC5297917 28177310

[bb31] Langan, P., Fisher, Z., Kovalevsky, A., Mustyakimov, M., Sutcliffe Valone, A., Unkefer, C., Waltman, M. J., Coates, L., Adams, P. D., Afonine, P. V., Bennett, B., Dealwis, C. & Schoenborn, B. P. (2008). *J. Synchrotron Rad.* **15**, 215–218. 10.1107/S0909049508000824 PMC2394804 18421142

[bb32] Langan, P. S., Vandavasi, V. G., Cooper, S. J., Weiss, K. L., Ginell, S. L., Parks, J. M. & Coates, L. (2018). *ACS Catal.* **8**, 2428–2437.

[bb33] Li, L., Shukla, S., Meilleur, F., Standaert, R. F., Pierce, J., Myles, D. A. A. & Cuneo, M. J. (2017). *Protein Sci.* **26**, 2098–2104. 10.1002/pro.3231 PMC5606543 28707382

[bb34] Livermore, D. M. & Woodford, N. (2006). *Trends Microbiol.* **14**, 413–420. 10.1016/j.tim.2006.07.008 16876996

[bb35] Matthews, B. W. (1968). *J. Mol. Biol.* **33**, 491–497. 10.1016/0022-2836(68)90205-2 5700707

[bb36] Matthews, B. W. (1976). *Annu. Rev. Phys. Chem.* **27**, 493–523.

[bb37] Meilleur, F., Munshi, P., Robertson, L., Stoica, A. D., Crow, L., Kovalevsky, A., Koritsanszky, T., Chakoumakos, B. C., Blessing, R. & Myles, D. A. A. (2013). *Acta Cryst.* D**69**, 2157–2160. 10.1107/S0907444913019604 24100333

[bb38] Moody, P. C. E. (1984). Thesis, Imperial College London.

[bb39] Murshudov, G. N. & Dodson, E. J. (1997). *CCP4 Newsl. Protein Crystallogr.* **33**, 31–39. http://www.ccp4.ac.uk/newsletters/newsletter33/murshudov.html.

[bb40] Myles, D. A. A., Dauvergne, F., Blakeley, M. P. & Meilleur, F. (2012). *J. Appl. Cryst.* **45**, 686–692.

[bb41] Nitanai, Y., Shimamura, T., Uchiyama, T., Ishii, Y., Takehira, M., Yutani, K., Matsuzawa, H. & Miyano, M. (2010). *Biochim. Biophys. Acta*, **1804**, 684–691. 10.1016/j.bbapap.2009.10.023 19883800

[bb42] Ohana, J., Jacquamet, L., Joly, J., Bertoni, A., Taunier, P., Michel, L., Charrault, P., Pirocchi, M., Carpentier, P., Borel, F., Kahn, R. & Ferrer, J.-L. (2004). *J. Appl. Cryst.* **37**, 72–77.

[bb43] Post, B., Schwartz, R. S. & Fankuchen, I. (1951). *Rev. Sci. Instrum.* **22**, 218–219.

[bb44] Romoli, F., Mossou, E., Cuypers, M., van der Linden, P., Carpentier, P., Mason, S. A., Forsyth, V. T. & McSweeney, S. (2014). *Acta Cryst.* F**70**, 681–684. 10.1107/S2053230X14005901 PMC4014346 24817737

[bb45] Schlichting, I., Berendzen, J., Chu, K., Stock, A. M., Maves, S. A., Benson, D. E., Sweet, R. M., Ringe, D., Petsko, G. A. & Sligar, S. G. (2000). *Science*, **287**, 1615–1622. 10.1126/science.287.5458.1615 10698731

[bb46] Schoenborn, B. P. & Langan, P. (2004). *J. Synchrotron Rad.* **11**, 80–82. 10.1107/s0909049503023902 14646140

[bb47] Skarżński, T., Moody, P. C. E. & Wonacott, A. J. (1987). *J. Mol. Biol.* **193**, 171–187. 10.1016/0022-2836(87)90635-8 3586018

[bb48] Stetten, D. von, Giraud, T., Carpentier, P., Sever, F., Terrien, M., Dobias, F., Juers, D. H., Flot, D., Mueller-Dieckmann, C., Leonard, G. A., de Sanctis, D. & Royant, A. (2015). *Acta Cryst.* D**71**, 15–26. 10.1107/S139900471401517X PMC4304682 25615856

[bb49] Teng, T.-Y. (1990). *J. Appl. Cryst.* **23**, 387–391.

[bb50] Teng, T.-Y. & Moffat, K. (1998). *J. Appl. Cryst.* **31**, 252–257.

[bb51] Tomanicek, S. J., Standaert, R. F., Weiss, K. L., Ostermann, A., Schrader, T. E., Ng, J. D. & Coates, L. (2013). *J. Biol. Chem.* **288**, 4715–4722. 10.1074/jbc.M112.436238 PMC3576076 23255594

[bb52] Vandavasi, V. G., Langan, P. S., Weiss, K. L., Parks, J. M., Cooper, J. B., Ginell, S. L. & Coates, L. (2017). *Antimicrob. Agents Chemother.* **61**, e01636-16. 10.1128/AAC.01636-16 PMC5192116 27795378

[bb53] Vandavasi, V. G., Weiss, K. L., Cooper, J. B., Erskine, P. T., Tomanicek, S. J., Ostermann, A., Schrader, T. E., Ginell, S. L. & Coates, L. (2016). *J. Med. Chem.* **59**, 474–479. 10.1021/acs.jmedchem.5b01215 26630115

[bb54] Wan, Q., Bennett, B. C., Wilson, M. A., Kovalevsky, A., Langan, P., Howell, E. E. & Dealwis, C. (2014). *Proc. Natl Acad. Sci. USA*, **111**, 18225–18230. 10.1073/pnas.1415856111 PMC4280638 25453083

[bb55] Wan, Q., Kovalevsky, A. Y., Wilson, M. A., Bennett, B. C., Langan, P. & Dealwis, C. (2014). *Acta Cryst.* F**70**, 814–818. 10.1107/S2053230X1400942X PMC4051544 24915100

